# Fusiform Correlates of Facial Memory in Autism

**DOI:** 10.3390/bs3030348

**Published:** 2013-07-05

**Authors:** Haley G. Trontel, Tyler C. Duffield, Erin D. Bigler, Alyson Froehlich, Molly B.D. Prigge, Jared A. Nielsen, Jason R. Cooperrider, Annahir N. Cariello, Brittany G. Travers, Jeffrey S. Anderson, Brandon A. Zielinski, Andrew Alexander, Nicholas Lange, Janet E. Lainhart

**Affiliations:** 1 Department of Psychology, University of Montana, Missoula, MT 59812, USA; E-Mail: htrontel@gmail.com; 2 Department of Psychology, Brigham Young University, Provo, UT 84604, USA; E-Mails: tduffield2009@yahoo.com (T.C.D.); erin_bigler@byu.edu (E.D.B.); 3 Neuroscience Center, Brigham Young University, Provo, UT 84604, USA; 4 The Brain Institute of Utah, University of Utah, Salt Lake City, UT 84112, USA; 5 Department of Psychiatry, University of Utah, Salt Lake City, UT 84112, USA; E-Mails: alyson.froehlich@hsc.utah.edu (A.F.); molly.prigge@hsc.utah.edu (M.B.D.P); jared.nielsen@hsc.utah.edu (J.A.N.); jason.cooperrider@hsc.utah.edu (J.R.C.); annahir.cariello@hsc.utah.edu (A.N.C.); 6 Department of Medical Physics, University of Wisconsin, Madison, WI 53706, USA; E-Mails: btravers@wisc.edu (B.G.T.); alalexander2@wisc.edu (A.A.); 7 Department of Radiology, University of Utah, Salt Lake City, UT 84112, USA; E-Mail: andersonjeffs@gmail.com; 8 Departments of Pediatrics and Neurology, Division of Child Neurology, University of Utah and Primary Children’s Medical Center, Salt Lake City, UT 84112, USA; E-Mail: brandon.zielinski@utah.edu; 9 Departments of Psychiatry and Biostatistics, Harvard University, Boston, MA 02138, USA; E-Mail: nlange@hms.harvard.edu; 10 Neurostatistics Laboratory, McLean Hospital, Belmont, MA, USA; 11 Waisman Laboratory for Brain Imaging and Behavior, University of Wisconsin, Madison, WI 53706, USA; E-Mail: jlainhart@wisc.edu (J.E.L.); 12 Department of Psychiatry, University of Wisconsin, Madison, WI 53706, USA

**Keywords:** autism, facial memory, fusiform gyrus, amygdala, hippocampus

## Abstract

Prior studies have shown that performance on standardized measures of memory in children with autism spectrum disorder (ASD) is substantially reduced in comparison to matched typically developing controls (TDC). Given reported deficits in face processing in autism, the current study compared performance on an immediate and delayed facial memory task for individuals with ASD and TDC. In addition, we examined volumetric differences in classic facial memory regions of interest (ROI) between the two groups, including the fusiform, amygdala, and hippocampus. We then explored the relationship between ROI volume and facial memory performance. We found larger volumes in the autism group in the left amygdala and left hippocampus compared to TDC. In contrast, TDC had larger left fusiform gyrus volumes when compared with ASD. Interestingly, we also found significant negative correlations between delayed facial memory performance and volume of the left and right fusiform and the left hippocampus for the ASD group but not for TDC. The possibility of larger fusiform volume as a marker of abnormal connectivity and decreased facial memory is discussed.

## 1. Introduction

Prior studies have shown that performance on standardized measures of memory in children with autism spectrum disorders (ASD) is substantially reduced in comparison to matched typically developing controls (TDC) [1,2,3,4,5,6]. This is not surprising given speculation of various white matter temporal lobe abnormalities in ASD [7] and the role that temporal structures play in memory and learning [8]. One measure that has demonstrated multiple differences in memory functioning in those with ASD compared to TDC [5,9] is the Test of Memory and Learning (TOMAL) [10]. The TOMAL is comprised of various verbal and non-verbal subtests, including the Facial Memory subtest. Performance on the TOMAL Facial Memory subtests may be of particular interest in studying memory impairments in ASD because of associated deficits in face processing [11] and atypicalities in the fusiform gyrus (For simplicity, throughout the paper we will often just refer to the fusiform and not fusiform gyrus, gyral, or volume.) of the temporal lobe [12]. Despite the large amount of research pertaining to facial processing in ASD, the literature examining facial memory is more limited, and a comprehensive understanding of facial memory functioning in this population is lacking [13,14,15]. 

### 1.1. Facial Processing and Memory as a Deficit in Autism

Facial processing has been recognized as a specific deficit in autism [16,17,18]. Individuals with ASD exhibit impairments in perception of facial affect [19,20,21], direction of eye gaze [22], eye contact [23,24], and attention to eyes [25,26]. Specific behavioral impairments in ASD help provide clues to the mechanisms of abnormal facial perception. For example, individuals with ASD tend to fixate longer on objects than faces (similar to typically developing individuals), but are less likely to scan regions of the face outside the primary facial features (*i.e.*, eyes, nose, mouth) [27,28,29,30]. Research also suggests that individuals with ASD may rely on individual features during facial processing rather than taking a holistic approach [31] and also may tend not to benefit from face orientation during facial recognition tasks [20,32]. These results provide evidence for atypicalities in how individuals with ASD attend to and process facial stimuli. 

Individuals with ASD have also been found to perform more poorly on facial recognition tasks relative to object recognition [13,14,33,34,35]. This has been suggested to be due to abnormal scanning of facial regions during encoding, which certainly could be the basis for impaired facial memory [29]. In a review of studies of facial perception in ASD, Weigelt *et al.* [36] reported that quantitative deficits in facial perception on behavioral tasks are much more impaired in tasks with memory demands, although other hypotheses have been proposed as well. For example, impaired facial perception may also be due to greater visuospatial effort required for facial processing [37], the inherent social content of face stimuli [38], or impaired gaze fixation [25,39]. Indeed, research on facial processing in ASD has revealed that individuals with ASD have impaired prototype formation of faces [11], which may help explain why once faces are attended to, they may be categorized and consolidated in memory very distinctly in ASD. 

Specific to facial memory, children with ASD are particularly impaired in their memory for faces [15], which may be less apparent in adolescence [14] and least apparent in adulthood [14,15]. Also, Kuusikko-Gauffin and colleagues [35] found that facial memory improved with age, with significant differences in facial memory between children, but not adolescents or adults. In addition, parents of ASD individuals had poorer facial memory performance than control parents.

### 1.2. Neuroanatomical Correlates of Facial Processing and Memory

Functional neuroimaging studies have identified a face-specific region in the fusiform gyrus of the temporal lobe termed the fusiform face area (FFA) [40]. The FFA is responsible for processing both facial features (e.g., nose, mouth, eyes), as well as the spatial relation among face parts [41,42,43,44,45,46,47]. Disruption of the fusiform face area in the fusiform gyrus may help explain why individuals with ASD have deficits in facial processing and facial memory. 

Kleinhans *et al.* [48], Anderson *et al.* [49], and Khan *et al.* [50] have shown reduced functional connectivity in ASD not only between the fusiform and other cortical areas but also between left and right fusiform gyri, and *within* the fusiform gyrus itself during face processing. Studies that have examined individuals with identifiable lesions to the fusiform have also demonstrated similar facial processing impairments [51,52,53]. Generally, the literature supports abnormalities in face-processing networks involving the fusiform, including reduced long-range and local functional connectivity (*i.e.*, within the fusiform face area) [50], rather than only a region specific abnormality [39,54,55]. 

The fusiform is also functionally related to both the amygdala and the hippocampus, two structures critical for memory and emotional processing. Studies looking at face processing, rather than memory, have found abnormal pathway microstructure [56] and connectively [50] between the hippocampus/amygdala and fusiform. All three of these regions show reduced activation during task-based fMRI studies of facial processing in autism [25,57,58,59]. Hypoactivation of the amygdala and fusiform is often observed in ASD individuals relative to TDC [55]. It is also important to consider that the lack of activation in these brain regions in individuals with ASD compared to TDC may also relate to differences in processing emotional intensity [60] and dynamic *versus* static facial stimuli [54,61]. Both facets of facial processing (*i.e.*, emotional intensity and dynamic expressions) have more ecological validity pertaining to social interaction deficits than the simple viewing of pictures of facial expressions (*i.e.*, static stimuli). Thus, a multitude of factors may influence face processing and facial memory. 

Several studies have examined fusiform gyral volume comparing controls to those with ASD. Volume in TDC individuals is considered a marker of structural integrity, albeit a coarse indicator of brain development [62]. ASD studies that have examined fusiform gyral volume have reported differences in size [63,64,65,66,67,68,69,70]. However, the direction of the differences, including hemispheric effects, varies. In a meta-analysis, Cauda *et al.* [71] reported a larger fusiform associated with autism, but underscored the variability of reported differences across studies. Inconsistencies in the volumetric literature in autism also exist for other temporal lobe structures such as the amygdala and hippocampus. In a sample of individuals with Asperger syndrome, Murphy *et al.* [72] found larger amygdala but not hippocampal volume. In contrast, Hasan, Walimuni and Frye [73] reported larger hippocampal volume in autism. The lack of a consistent direction to volume differences in the fusiform may be a reflection of the heterogeneity of ASD and associated morphological differences that may also result in varied cognitive impairments. 

Additionally, variability in volumetric findings of temporal lobe structures in ASD likely has to do with age, developmental, and maturation effects. For example, some volumetric studies have implicated early overgrowth followed by normalization of amygdala volumes in middle childhood with either normalization or persistence of hippocampal enlargements [56]. Some studies only examined adults, like Dziobek, Bahnemann, Convit, and Heekeren [68], who found that the relationship between amygdala volume and fusiform thickness was actually smaller in autism compared with TDC, providing further evidence for disrupted neural networks. Still others have found reduced volume of the hippocampal–amygdala complex in autism in adolescents and adults [63,74]. As such, variability in reported volumetric findings of temporal lobe structures in ASD likely reflects differences in age and heterogeneity of the disorder. Pelphrey, Shultz, Hudac, and Vander Wyk [55] propose that “ASD begins with a failure in the emergence of the specialized functions of one or more of the set of neuroanatomical structures involved in social information processing. This failure happens early in ontogeny, within the first nine months to one year of life, if not earlier. In turn, because the affected regions do not generate the normal stream of both intrinsic and stimulus driven signals, the normal developmental pattern of connections among these brain regions is greatly altered” (p. 4). Thus, examining volumetrics, although not a direct measure of neural connectivity, represents a logical place to begin in understanding how it might influence abnormal functioning of specialized neuroanatomical structures (and subsequently connectivity and functionality). 

How fusiform morphology may contribute to impairments in facial memory is not yet known. However, it would seem to be a logical structure for examination, given the role the fusiform plays in face processing. Likewise, because of the important role that the medial temporal lobe plays in memory—particularly the hippocampus and, to a certain extent, the amygdala—it would be important for any facial memory study to volumetrically assess these regions, as well. Accordingly, the current study investigated whether hippocampal, amygdala, or fusiform gyral volume related to performance on the TOMAL Facial Memory task, both immediate and 30-minute delayed recall, in children 5 to 19 years of age with ASD compared to TDC age-matched individuals. The format for assessing TOMAL Facial Memory includes an immediate recognition recall trial where previously observed faces have to be identified amidst foils not seen. With each trial, the number of target faces and foils increases. Because performance on this initial trial requires face processing, individuals with ASD would be expected to perform more poorly, possibly just because of the challenges specific to processing facial information.

The delayed recognition trial occurs 30 min after the immediate recall trial and is composed of faces that have been previously viewed along with foils that have not. The child has no opportunity for rehearsal during the 30-min interval. Since the previously seen face has already been initially processed, this delayed aspect of the TOMAL Facial Memory Task taps consolidation. As a contrast to facial memory, the TOMAL also utilizes visual memory tasks such as Visual Selective Reminding that has no aspect of face processing but rather visual spatial retention, using both immediate and delayed recall. Also, the Object Recall task assesses immediate retention of visually presented line drawings of common objects including a single drawing of a generic face as one of 24 stimuli. Object recall does not have a delayed retention measure. By comparing ASD and TDC participants on the TOMAL Facial Memory subtest with visually processed memory tasks like the Visual Selective Reminding and Object Recall provides the comparison of how specific an impairment in facial memory may be or whether more general non-verbal, visual memory impairments may be associated with ASD. Also, of importance is whether these TOMAL memory measures relate to fusiform, hippocampal and amygdala volume. 

We examined several hypotheses about the role of fusiform gyral, hippocampal, and amygdala volume in TOMAL Facial Memory performance. First, it was hypothesized that facial memory performance would be significantly lower for individuals with autism than for TDC participants. Second, given the literature supporting volume differences in these temporal lobe structures in ASD, it was hypothesized that the fusiform gyral, amygdala, and hippocampal volumes would be larger for the ASD group when compared with TDC participants and, furthermore, that these structures would be negatively correlated with TOMAL Facial Memory performance, both immediate and with a 30-min delay; however, that fusiform gyral volume would not correlate with TOMAL Performance on the Visual Selective Reminding and Object Recognition. 

## 2. Method

### 2.1. Ascertainment

Autism and TDC participants were recruited predominantly from community sources, including parent support groups, youth groups, and schools, and from clinic social skills groups, as described by Bigler *et al.* [75] and Alexander *et al.* [76]. The subjects in this study are a subset of participants in a longitudinal investigation of late brain development from three years of age through early adulthood. The subset for this investigation was selected from the larger sample based on age within the reference norms of the TOMAL, having complete TOMAL data from the time of initial assessment, and closeness of group matching on age, PIQ, handedness, and head circumference. All facets of this investigation were undertaken with the understanding and written consent of each subject or legal guardian, with the approval of the University of Utah and Brigham Young University Institutional Review Boards, where testing was performed, and in compliance with national legislation and the Code of Ethical Principles for Medical Research Involving Human Subjects of the World Medical Association.

### 2.2. Subject Groups

All subjects were males, 5–19 years of age. The ASD group had a total of 56 participants and the TDC group a total of 31 participants with complete neuropsychological and neuroimaging datasets. Potential sex differences in memory were not examined. 

### 2.3. Idiopathic Autism Sample

Autism was diagnosed rigorously. The subject’s parent was interviewed using the Autism Diagnostic Interview–Revised (ADI-R) [77], a semi-structured, investigator-based interview with good reliability and validity. All subjects with autism were also directly assessed using the Autism Diagnostic Observation Schedule–Generic (ADOS-G) [78], a semi-structured play and interview session designed to elicit social, communication, and stereotyped repetitive behaviors characteristic of autism. All autistic subjects met ADI–R, ADOS–G, and the *Diagnostic and Statistical Manual of Mental Disorders–Fourth Edition* (DSM–IV) criteria for autistic disorder [79]. History, physical exam, fragile X gene testing, and karyotype, performed on all subjects, excluded medical causes of autism. In regards to medications, fifteen individuals with ASD were on psychotropic medications, including five participants on stimulant medications. None of these individuals represent outliers in the data. 

### 2.4. Control Sample

Typically developing control subjects had no developmental, neurological, or clinical history of major psychiatric disorders. Control subjects likewise completed an assessment with the ADOS-G and were assessed rigorously for autism spectrum disorders to ensure none met any criterion. Two participants in the control group were on allergy medications.

### 2.5. IQ

In the current study, IQ was used as a selection and descriptive variable, to ensure that both controls and ASD participants met the criterion. Because of age differences at the time of recruitment, different versions of intellectual tests were used over the 10 years of subject accrual to the parent project. Summary IQ findings were based on one of the following: *Wechsler Intelligence Scale for Children–Third Edition* (WISC–III), *Wechsler Adult Intelligence Scale–Third Edition* (WAIS-III), *Wechsler Abbreviated Scale of Intelligence* (WASI; VIQ and PIQ indexes) [80,81,82], or *Differential Ability Scales* (DAS) [83]. IQ was not used as a covariate because IQ and memory performance are highly interrelated making it an inappropriate covariate in neurodevelopmental study such as this because it would over-control the dependent memory variable [84]. 

### 2.6. Head Circumference and Handedness

No unusual developmental anomaly was found in the sample that may relate to cognitive outcome [85]. Macrocephaly occurs with a greater frequency in autism for ~20% of children [86]; the control sample was group-matched for head circumference. Handedness was measured using the Edinburgh Handedness Inventory [87]. A score of +100 signifies complete right-handedness and –100 indicates complete left-handedness.

### 2.7. Neuroimaging

Volumetric studies were based on magnetic resonance images acquired on a Siemens Trio 3.0 Tesla scanner at the University of Utah. A 12-channel RF head coil was used to obtain 3D T1-weighted image volumes with 1 mm isotropic resolution using an MP-RAGE sequence (TI = 900 msec, TR = 2300 msec, TE = 2.91 msec, flip angle = 9 degrees, sagittal, field of view = 25.6 cm, matrix = 256 × 256 × 160).

### 2.8. Volumetric Image Analysis

All analyses were performed with FreeSurfer, version 5.1 (http://surfer.nmr.mgh.harvard.edu/), following the methods detailed by Bigler *et al.* [88], and included automated volume calculations of the following temporal lobe regions of interest (ROI): fusiform gyrus, amygdala and hippocampus along with total intracranial volume (TICV). It should be noted that hippocampus segmentations are more reliable than amygdala segmentations [89]. TICV was used as a matching variable and covariate. 

#### 2.8.1. Memory

Although the entire TOMAL was administered, and generally samples various domains of memory in children and adolescents, ages 5 years 0 months through 20 years 0 months, this study focused specifically on the Facial Memory subtest [10]. Details of overall TOMAL performance in autism have been reported by Southwick *et al.* [5]; however, that study did not explore any brain correlates. The Facial Memory subtest was administered according to standard methods with delayed retention assessed at 30 min after the immediate recognition trials. The Facial Memory subtest is a nonverbal subtest requiring recognition and identification of previously viewed faces from a set of distracters: black-and-white photos of various ages, males, and females, and various ethnic backgrounds. There is an immediate recognition score, as well as a 30-min delayed score. Visual Selective Reminding is a nonverbal free-recall task in which the examinees point to specified dots on a card, after a demonstration of the examiner, and are reminded only of items recalled incorrectly. Trials are continued until mastery is achieved or through eight trails. Visual Selective Reminding also has a delayed recall task after 30 min. During Object Recall, the examinees are presented with a series of named pictures and have to recall them across four trials. There is no delayed portion of the Object Recall subtest. 

#### 2.8.2. Statistical Analysis

Multivariate analysis of covariance (MANCOVA) was employed to describe group means for autism and control subjects on the TOMAL, with TICV as a covariate for volumetric analyses. Additionally, MANCOVA was employed to describe group means for autism and control subjects on volumetric analyses, with TICV and age as covariates. Lastly, partial correlations were run to examine the relationship between TOMAL performance and ROI volume, controlling for age and TICV. 

## 3. Results

### 3.1. Sample Characteristics

As shown in Table 1, no significant differences were found between groups for group-matching variables (age, head circumference, handedness index) except for IQ. 

**Table 1:** Demographic Information.

	ASD (*n* = 56)			Typically-developing (*n* = 31)				
	Mean	SD	Range	Mean	SD	Range	*t*	*p*
Age in years	12.00	4.37	5.00–19.75	11.98	4.01	5.25–19.33	0.02	0.98
Head Circumference (cm)	54.71	5.81	50.70–60.50	55.34	2.13	51.80–60.50	–0.00	0.99
Total Intracranial Volume (TICV,cm3)	1672.32	157.84	1268.66–2063.84	1675.94	176.45	1399.97–2171.21	0.10	0.92
Handedness Inventory	61.45	54.78	–100–100	65.28	44.97	–80–100	0.01	0.99
Wechsler FIQ	98.26	16.63	61–137	115.24	15.57	87–152	4.53 **	< 0.001
Wechsler PIQ	102.41	16.00	66–138	116.13	15.46	90–155	3.88 **	< 0.001
Wechsler VIQ	95.57	20.92	55–145	110.94	16.14	74–140	3.54 **	< 0.001

* = *p <* 0.05; ** = *p* < 0.01.Edinburgh Handedness Inventory on a scale from –100 (left-handed) to 100 (right-handed).

### 3.2. Facial Memory Performance in Autism and Controls

As shown in Table 2, a MANCOVA revealed that individuals in the autism group performed significantly worse on the Facial Memory subtest F(1, 82) = 32.76, *p* < 0.01 and the Facial Memory Delayed subtest F(1, 82) = 14.32, *p <* 0.01. ASD participants also performed significantly more poorly on the Visual Selective Reminding immediate and delayed recall, a task with only abstract visual stimuli, as well as with the Object Recall, which had only a simple line drawing of a single face amongst numerous other common and familiar objects. 

To insure that memory performance differences were related to autism and not simply to generalized lower cognitive functioning, we individually matched (±7 points) participants with autism to typical developing controls on IQ. All TOMAL subtests remained significantly different between the groups even after IQ matching.

**Table 2:** Mean Scaled Score TOMAL Performance by Group (MANOVA).

Measure	Mean (SD) ASD *n* = 56	Mean (SD) TDC *n* = 31	F	*p*	ρη^2^
Facial Memory	7.29 (2.36)	10.52 (2.53)	32.76	< 0.001	0.28
Facial Memory Delayed	7.64 (2.73)	9.83 (2.17)	14.32	< 0.001	0.15
Object Recall	5.98 (3.45)	9.37 (2.70)	21.57	< 0.001	0.21
Visual Selective Reminding (Immediate)	7.48 (3.30)	9.87 (2.56)	11.76	< 0.001	0.12
Visual Selective Reminding (Delayed)	8.75 (2.03)	10.10 (1.52)	10.06	< 0.001	0.11

Note: TOMAL subtest scores are age-corrected scaled scores. ρη^2^ = partial eta squared. Partial eta squared is an effect size measure that shows the variance explained by the predictor (TOMAL subtest) after excluding variance explained by other predictors (TICV).

### 3.3. ROI Volumes in Autism and Controls

MANCOVA was used to compare facial memory ROI volumes (Table 3), including the left and right fusiform gyrus, amygdala, and hippocampus. Upon controlling for TICV, the left amygdala and the left hippocampus were significantly larger in ASD than in controls, while the left fusiform gyrus was significantly larger in controls. Effect sizes were minimal and no other volume differences were found between the groups. 

**Table 3:** ROI volume multivariate analysis controlling for TICV and Age.

Structure	Mean (SD) ASD *n* = 56	Mean (SD) TDC *n* = 31	F	*P*	*ρη^2^*
center Fusiform	12.12 (1.89)	12.95 (2.03)	4.91 *	0.03	0.06
Right Fusiform	11.81 (1.79)	11.64 (1.67)	0.25	0.62	0.00
center Amygdala	1.78 (0.30)	1.67 (0.24)	4.07 *	0.049	0.05
Right Amygdala	1.78 (0.28)	1.72 (0.23)	1.21	0.27	0.01
center Hippocampus	4.62 (0.61)	4.39 (0.66)	4.15 *	0.048	0.05
Right Hippocampus	4.61 (0.65)	4.56 (0.46)	0.40	0.53	0.01

Note: ROI volumes are measured in centimeters cubed. TDC = typically-developing controls; ASD = Autism Spectrum Disorder. * = *p <*0.05. ρη^2^ = partial eta squared. Partial eta squared is an effect size measure that shows the variance explained by the predictor (TOMAL subtest) after excluding variance explained by other predictors (TICV, age).

### 3.4. Relationship between ROI Volume and Facial Memory Performance

Using partial correlations controlling for TICV (see Table 4), the Facial Memory subtest was not significantly correlated with any ROI volume. However, Facial Memory Delayed was significantly negatively correlated with left fusiform gyrus and right hippocampal volume (*r* = –0.29, *p* = 0.042 and *r* = –0.28, *p* = 0.046, respectively) in the autism group, indicating that as left fusiform gyrus and right hippocampal volumes increased, performance on the delayed facial memory decreased. TOMAL Facial Memory performance (immediate and delayed) was not significantly correlated with any ROI volume in controls. 

**Table 4:** Partial Correlations between facial memory ROI volumes and TOMAL performance–controlling for TICV and age.

Structure	Facial Memory		Facial Memory Delayed	
	*ASD*	*TDC*	*ASD*	*TDC*
center Fusiform	0.10	0.05	–0.28 *	–0.21
Right Fusiform	0.13	0.12	–0.15	–0.09
center Amygdala	0.04	–0.01	–0.02	0.09
Right Amygdala	0.07	–0.04	–0.11	–0.07
center Hippocampus	–0.05	0.06	–0.11	0.08
Right Hippocampus	–0.17	0.17	–0.28 *	0.14

Note: The correlations presented are Pearson’s r scores. ASD = Autism Spectrum Disorder; TDC = typically-developing controls; * = *p <* 0.05.

To test the specificity of the observed significant relationship between Delayed Facial Memory and fusiform gyrus volume, ROI volume comparisons were subsequently performed for Object Recall and Visual Selective Reminding, immediate and delayed, as shown in Table 5. No significant correlations were observed (*p* > 0.05).

**Table 5:** Partial correlations between facial memory ROI volumes and TOMAL performance—controlling for TICV and age.

Structure	Object Recall		Visual Selective Reminding (Immediate)		Visual Selective Reminding (Delayed)	
	*ASD*	*TDC*	*ASD*	*TDC*	*ASD*	*TDC*
Left Fusiform	0.08	–0.08	0.13	–0.24	0.11	0.13
Right Fusiform	–0.01	0.01	–0.14	–0.07	0.10	0.26
Left Amygdala	–0.14	0.01	–0.13	–0.11	0.06	0.10
Right Amygdala	0.03	–0.04	–0.14	–0.14	0.14	0.12
Left Hippocampus	–0.20	0.19	–0.02	–0.18	0.07	–0.16
Right Hippocampus	–0.16	0.17	–0.24	–0.18	0.08	0.26

Note: The correlations presented are Pearson’s r scores. ASD = Autism Spectrum Disorder; TDC = typically-developing controls.

## 4. Discussion

**Figure 1:**
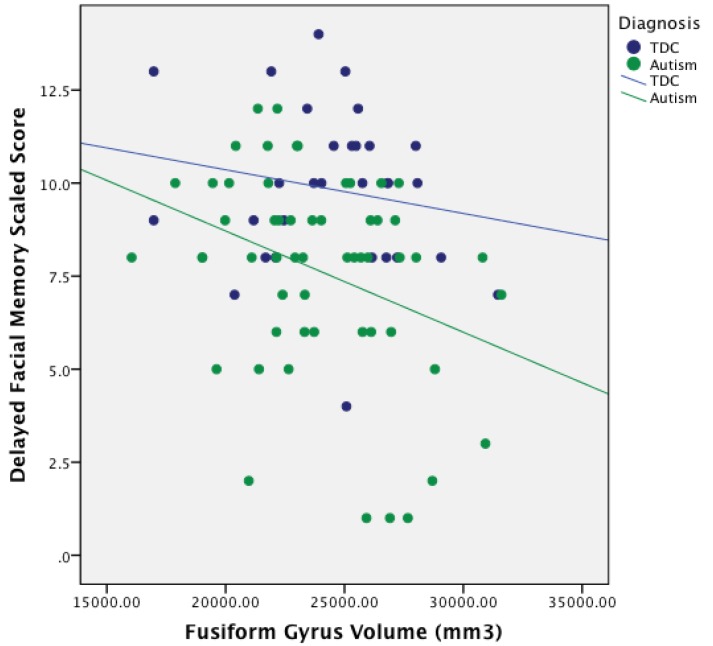
Relationship between Fusiform Gyrus Volume and Delayed Facial Memory Performance in ASD.

Note: TDC = Typically Developing Controls. mm3 = millimeters cubed.

**Figure 2:**
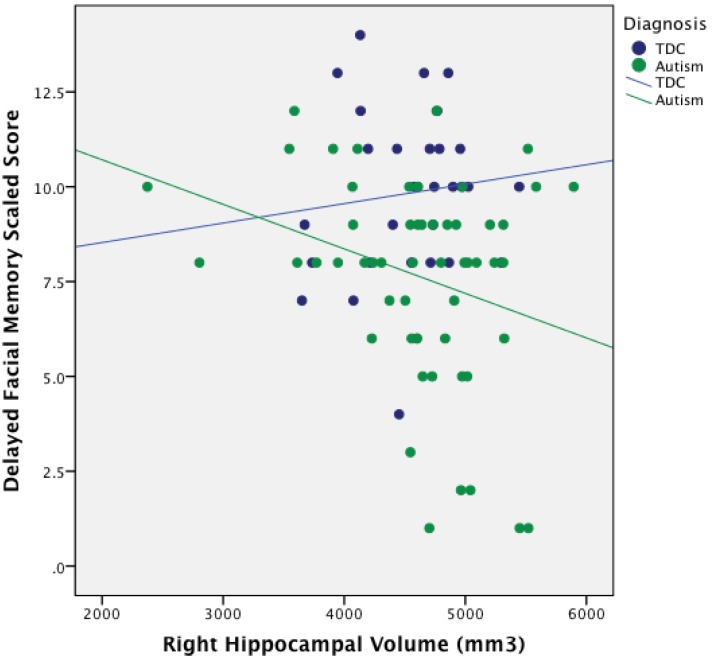
Relationship between Right Hippocampal Volume and Delayed Facial Memory Performance in ASD

Note: TDC = Typical Developing Controls. mm3 = millimeters cubed.

Anatomically, ASD participants had significantly smaller left fusiform but larger left amygdala and hippocampal volumes compared to TDC participants. With regards to memory performance, ASD participants performed significantly lower than TDCs on the TOMAL Facial Memory, Object Recall, and Visual Selective Reminding Test, consistent with other studies that have reported similar memory deficits in autism [1,2,5,6]. However, only the delayed component of the TOMAL Facial Memory test related to fusiform and hippocampal volume (see Figure 1). Interestingly, the fusiform correlation with Facial Memory was negative and significant only on the left and only for the delayed component. The significant hippocampal volume relation with delayed Facial Memory was also negative, but only on the right (see Figure 2). As can be seen in Figure 1, all but one of the participants with the largest fusiform volumes *and* poorest Delayed Facial Memory scores were in the ASD group. Likewise, those ASD participants with the lowest performance on the delayed Facial Memory task had the largest right hippocampal volumes. This association of larger size of the fusiform and hippocampus with poorer consolidation of facial memory implicates aberrant development of these structures in ASD, but only within a subset of individuals with ASD.

A number of functional neuroimaging studies have shown that the working memory aspect of face processing and immediate retention involves bilateral fusiform and hippocampal areas [90,91]. However, given the generally non-verbal nature of facial memory, more right ventral and medial temporal lobe participation may be involved [92,93,94,95]. Given the potential rightward bias for facial memory, the larger right-sided hippocampus findings in ASD being associated with poorer delayed TOMAL Facial Memory performance appears somewhat straightforward. Larger size may reflect neural irregularities within the hippocampus that are associated with impaired face memory. Explanation for why larger size of only the *left* fusiform was associated with poorer delayed Facial Memory is perplexing and requires more inference. The TOMAL Facial Memory task is considered part of the non-verbal core of memory tasks but there are no subject restrictions that would preclude the participant from using verbal cues (*i.e.*, that face is round with a small mouth) in performing the task, which may be a factor in the relation between left fusiform volume and facial memory performance. In fact, Grossman, Klin, Carter, and Volkmar [130] found that when ASD children are presented with facial emotional processing tasks, they perform better with words for emotions rather than faces. Also, Brown and Lloyd-Jones [96] have shown that subjects who verbally describe a face before performing the non-verbal face recognition task performed better. As a group, the ASD subjects had *smaller* overall fusiform volume, but it was its larger size that related to poorer consolidation of face memory. 

### 4.1. Fusiform Gyrus Volumes in Autism

Abnormal size of the fusiform gyrus in autism has been previously reported as reviewed in the introduction [63,64,65,66,67,68,69,70]. In the current study, only the left fusiform was significantly smaller. Using a voxel-based morphometry technique, Toal *et al.* [67] observed that individuals with autism had decreased volumes in medial temporal and fusiform gyrus regions relative to controls. Reduced fusiform gyral volumes may be related to age as evidenced by Wallace *et al.* [69] who found thinner fusiform gyrus cortex in autism that appeared to increase with age. Raznahan *et al.* [70] also found a relationship between size and age, revealing smaller cortical volumes and thickness in fusiform and middle temporal gyri in children with ASD. Similarly, van Kooten *et al.* [97], in a post-mortem histological study involving 7 individuals with ASD, found fewer and smaller neurons in the fusiform gyrus in autism. How neuronal count actually relates to neuroimaging measured volume is not known at this time. However, this may be a key area for future study. In contrast, using voxel-based morphometry, Waiter *et al.* [64] found right-side fusiform gyral volume to be significantly larger. Likewise, in adults, larger fusiform gyral volumes were found in ASD [70], and still other studies have found no anatomical differences in fusiform gyrus volumes associated with autism [63]. Regardless of these differences and inconsistencies related to fusiform volume and ASD, the TDC and ASD participants in this sample did not differ in age yet, as shown in Figure 1, larger fusiform on the left was associated with poorer consolidation of facial memory. 

While not understood at this time, one reason for neuroanatomical variability in autism may largely be due to the clinical and genetic heterogeneity of ASD and cross-sectional age-related developmental differences in neuroanatomical size between childhood and adulthood. Variability is also expected because of different methods used in volume quantification. Possibly one key to understanding this rather confusing picture about fusiform volume, development, and ASD may be that volumetric differences, regardless of direction, exist within these medial and ventral temporal lobe structures in ASD that reflects heterogeneity of fusiform development. Clearly, these regions participate in memory, social cognition, and face processing [64] but also dynamically change with maturation [70]. Thus, the answer for what fusiform volume and laterality may mean in facial memory likely will require prospective, longitudinal investigations. 

### 4.2. Atypical Structure–Function Relationship of Fusiform Gyrus Morphometry in Autism

When attempting to interpret structure- and size-function relations, there has been much discussion across all of biology as to a possible “Goldilocks” effect—just the right size or amount for maximal function [98,99,100]. As such, optimal size–function relations are often reflected in positive associations [101]. In partial support for the “bigger is better” hypothesis, Gautam *et al.* [102] examined structure–function relations that differ by age. In aging, larger cortical volumes often equate to larger remaining portions of functional neural substrates (*i.e.*, brain reserve) and hence lead to better functionality and resistance to the effects of age-related degeneration and onset of neurodegenerative disorders. However, in autism, larger development may signal early overgrowth—an indication of aberrant connectivity. Overgrowth provides no advantage as has been shown in aging and neurodegenerative studies of non-ASD individuals [103,104,105]. Thus positive correlations between volume loss and neuropathology, such as in Alzheimer’s and mild cognitive impairment (MCI) [102,106,107], have implications about cognitive functioning that are vastly different than the relationship between size and function in typical child development and ASD [108]. As can be seen in both Figure 1, Figure 2 the negative correlations were driven by a subset of ASD subjects with the largest volumes yet poorest Facial Memory scores. A number of ASD participants had Facial Memory scores and fusiform and hippocampal volumes that completely overlapped with TDC participants, underscoring that this larger size relationship with poorer memory represents yet another heterogeneous finding in ASD.

Interestingly, although facial memory was not specifically examined, Dziobek, Bahnemann, Convit, and Heekeren [68] observed that a region of increased cortical thickness within the fusiform gyrus was associated with impairments in face processing in autism. There is some speculation that these aberrant neural growth patterns in autism may reflect some failure in cellular pruning, which may be regionally manifested [109]. 

In our sample of ASD participants, it is possible that the larger size of the fusiform in a subset reflects regional abnormalities in pruning with associated errors in connectivity and this is why some ASD subjects (as shown in Figure 1) who had the largest fusiform volume also had the poorest consolidation of memory for faces. Overall, a failure in growth regulation (whether it be undershooting or overshooting), is a perplexing factor associated with autism and may be why greater size is related to poorer function in this sample of individuals with ASD. Brain growth rate abnormalities have been inferred in ASD [110,111], and would fit with the negative correlation between size and performance in ASD. These abnormalities likely reflect disrupted developmental neurobiology in ASD, and bolsters the notion that size-function relationships in ASD are an important piece to understanding the disorder. Indeed, dysfunction in one brain region likely affects development and functioning of related brain regions, leaving complex and individualized neurodevelopmental patterns among ASD individuals. 

With regards to memory in general, the fusiform has been implicated as an important region in visual memory consolidation processes [112,113]. Specific to face-location and face-name associations, functional neuroimaging studies have demonstrated that the region of the fusiform gyrus plays a role in memory consolidation [91,114]. Consistent with its putative role in consolidation, the current findings suggest that larger fusiform volume uniquely affects consolidation of face memory in ASD. 

### 4.3. Relationship between Structure Size and Connectivity

How can evidence of the pathology of increased size be reconciled with evidence of pathological functional under-connectivity both within (short-range) and between (long-range) brain structures in autism [50]? Courchesne and Pierce [115] have outlined some of the morphological differences seen in the developing brain in autism, where altered developmental trajectories may result in different volumes depending on age; differences in volume likely reflect different organization within a structure as well as its connectivity with other structures [116]. Likewise, any disruption of early brain development, even if a given structure eventually normalizes in volume with age, has the potential to significantly influence circuitry and resultant function [117]. The relation between abnormal developmental brain growth trajectories and abnormal connectivity in autism has been implicated in a number of studies [118,119], with disrupted connectivity representing major theories of autism [115,117,120,121,122,123,124]. Hazlett *et al.* [119] observed larger temporal lobe volume in young ASD subjects out to age 6. Although they did not specifically assess the fusiform gyrus, lobular volume is typically highly positively related to individual gyral volumes [88]. Gyral volumes outside of some optimal size may be an indicator of abnormal connectivity, helping to explain the negative correlation between fusiform volume and delayed facial memory performance in the current study. Additionally, Casanova *et al.* [125,126,127] have shown that increases in cortical gray matter may relate to aberrant increases in white matter via increased white matter projections necessary to maintain the connectivity of the increased number of cortical cells in the autistic brain. However, increased number of white matter connections in autism may not result in greater and more efficient functional connectivity. 

Given the research suggesting that facial processing may change with age in typical individuals [128], and cross-sectional age-related research suggesting atypical trajectories of fusiform volume and cortical thickness in autism [70,71], examination of facial memory from a longitudinal approach will hopefully answer some of these questions. Additionally, given the potential importance of the rapid subcortical face detection and attention system [18], as well as the cortical face processing system involving the fusiform gyrus in autism, coordinated longitudinal multimodal examination of both systems may help discern causal pathways in brain development leading to impaired face processing. Given the current findings, it may be especially important to examine consolidation of face memory in relation to face processing and brain development. One can only imagine the extraordinarily negative impact impaired facial memory has on understanding and navigating the social world across the lifespan. As such, unraveling the neuroanatomical basis to face processing and facial memory impairments in autism represents an important topic of investigation. 

As discussed in the introduction, the fusiform, hippocampus and amygdala are all connected both intra- and inter-hemispherically. A major limitation of the current investigation is that only volumes of these structures were examined and not their functional connectivity with regards to facial memory. As Frith [130] has pointed out, only limited inferences can be made when only one dimension—like volume—of a neural system is examined in disorders such as autism. Accordingly, a next step in this line of research will need to not only explore volumes of these regions but additional indicators of their morphology and functional connectivity in retention of facial memory.

## 5. Conclusions

The objective of the current study was to examine facial memory in autism and to explore the relation of facial memory performance with anatomical ROI volumes known to be involved in face processing. Larger volumes in the autism group in the left amygdala and left hippocampus compared to TDC were found in this sample. In contrast, TDC had larger left fusiform gyral volumes when compared with ASD. These differences did not relate to TOMAL Facial Memory for the immediate trials but negative correlations between delayed Facial Memory performance and the left fusiform and right hippocampus for the autism group but not for TDC were found. It is possible that larger fusiform gyrus and hippocampal volumes may be a marker of abnormal connectivity and functionality in facial memory.
